# Visible Light-Responsive Photocatalytic Activity of Boron Nitride Incorporated Composites

**DOI:** 10.3389/fchem.2018.00440

**Published:** 2018-09-24

**Authors:** Ning Wang, Guang Yang, Haixu Wang, Rong Sun, Ching-Ping Wong

**Affiliations:** ^1^Shenzhen Institutes of Advanced Technology, Chinese Academy of Sciences, Shenzhen, China; ^2^Department of Electronics Engineering, The Chinese University of Hong Kong, Hong Kong, China

**Keywords:** photocatalysts, boron nitride, composites, visible light, charge transfer, hole-electron recombination

## Abstract

Photocatalysts are essential to promote the highly efficient applications of solar energy in water splitting and/or the degradation of organic contaminations. Especially, the visible light-responsive photocatalysts could benefit with the cost-effective splitting or degradation due to the unlimited sunlight and the absence of expensive light emitter. In the photocatalysts, the charge transfer rates as well as the hole-electron recombination rate are two critical factors that determine the photocatalytic activity, which could also be affected by the dimension, defects, doping and morphologies controlled by the synthesis methods. Boron nitride (BN) is an ultrawide-bandgap semiconductor, and the combination of BN with the visible light-responsive photocatalysts has been found to be effective in enhancing the photocatalytic activities. Therefore, it should be meaningful to understand the BN incorporated photocatalytic composites in depth, including the synthetic approaches, the activity improving mechanisms and the versatile applications. In this review, we mainly focused on the assembly method of BN incorporated photocatalysts; the activity enhancing mechanism by introducing the BN in the photocatalytic composites as well as the properties and the applications. In the end, we gave a conclusion and an outlook for the BN incorporated photocatalytic composites.

## Introduction

Photocatalysts are the semiconductor materials that could promote the water splitting and the degradation of organic contaminations via converting the light energy of irradiation to the chemical energy (redox reaction) of hole-electron pairs, which has attracted intense interests for decades (Molinari et al., 2010). The most impressing advantage of photocatalysts is the utilization of unlimited sunlight on the earth, ranging from the ultraviolet to visible light (Zhu and Wang, 2017; Di et al., 2018; Guo et al., 2018; Jiang et al., 2018; Ji M. et al., 2018; Liang et al., 2018; Li et al., 2018; Nasr et al., 2018; Sivaprakash et al., 2018; Xue et al., 2018), which benefits the human beings with an alternative approach to get the H_2_/O_2_ and self-cleaning in an environment friendly way.

The activities of the photocatalysts were mainly determined by three factors: (a) the light absorption, (b) the mobility of light-generated carriers, and (c) the recombination rate of the hole-electron carriers. The light absorption was mainly dependent on the bandgap of the photocatalysts. For example, TiO_2_ (*E*_g_ = 3.0–3.2 eV)/SnO_2_ (*E*_g_ = 3.8 eV)/ZnS (*E*_g_ = 3.7 eV)/ZrO_2_ (*E*_g_ = 5.0 eV) could be used as the ultraviolet light-responsive photocatalysts, while the WO_3_ (*E*_g_ = 2.8 eV)/CdS (*E*_g_ = 2.5 eV)/GaP (*E*_g_ = 2.3 eV)/V_2_O_5_ (*E*_g_ = 2.0 eV)/CdSe (*E*_g_ = 1.7 eV)/GaAs (*E*_g_ = 1.4 eV) could be used as the visible light-responsive photocatalysts (Molinari et al., 2010). Besides, the surface plasmon resonance (Zheng et al., 2011; Rayalu et al., 2013) as well as the heterostructure design (Xie et al., 2017; Xu et al., 2017a,b) could also improve the light absorption. The mobility of the hole/electron carriers in the photocatalysts was relevant to the conductivity of the photocatalysts (Tang and Ye, 2005; Xu et al., 2012; Crossland et al., 2013) and the effective mass of the carriers (Zhang J. et al., 2015). On the other hand, the higher mobility could facilitate the well-separation of the hole/electron pairs (Zhang J. et al., 2015), and thus improve the photocatalytic activities.

Hexagonal boron nitride (BN) is an analog of graphite, characterized with a layered structure. BN has an ultra-wide bandgap (5–6 eV), which benefits the material with high electrical insulation, high thermal and chemical stability (Tsao et al., 2018). The as-synthesized BN nanostructures were always negatively charged intrinsically (Peng et al., 2013), which makes them the good *h*^+^ carrier acceptor, and could be used to improve the *h*^+^/e^−^ carrier separation in photocatalysis. As reported in literatures (Chen et al., 2014; Wang J. et al., 2015; Štengl et al., 2016; Liu et al., 2017; Li et al., 2018; Nasr et al., 2018), the combination of BN in the photocatalysts could effectively enhance the photocatalytic activities via improving the visible light absorption and reducing the recombination rate of hole/electron carriers. Therefore, the BN incorporated nanocomposite could be an alternative photocatalyst with enhanced visible light-responsive photocatalytic activities.

This work aims to review the recent progress of visible-light responsive BN incorporated photocatalysts, including the methodology for heterostructure assembly, characterization, and the activity enhancing mechanisms. Finally, several approaches will be proposed for further improving the photocatalytic activities of the BN incorporated nanocomposites.

## Assembly of BN incorporated nanocomposites for photocatalysis

The assembly of BN incorporated photocatalysts could be accomplished by hydrothermal, ball-milling, calcined, electrospinning, *in-situ* precipitation, impregnation, microwave-assisted, and water bath methods.

### Hydrothermal synthesis

**Figure 1:**
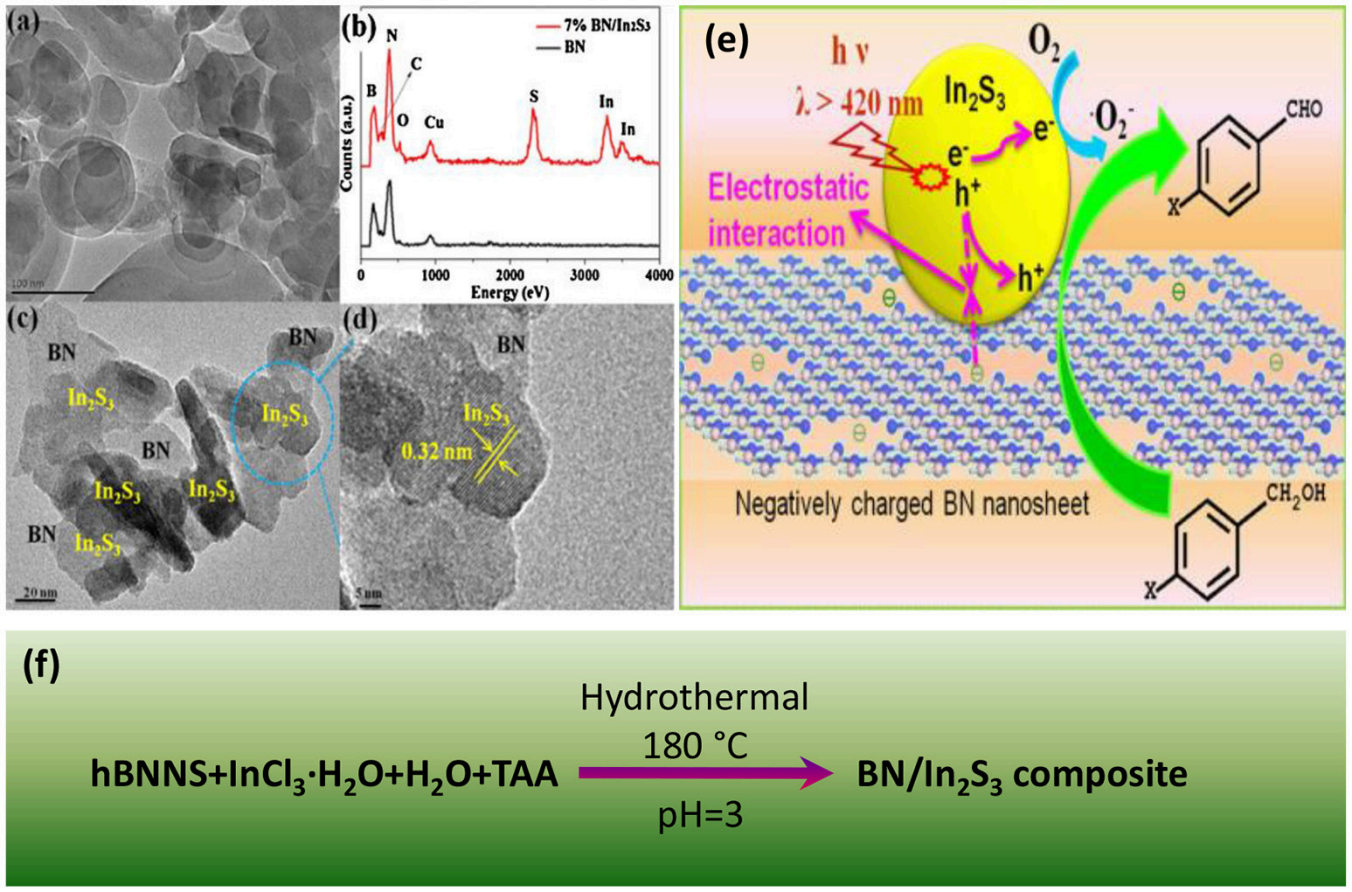
**(a)** TEM image of BN nanosheets. **(b)** EDX patterns of In_2_S_3_ and 7% BN/In_2_S_3_. **(c)** TEM and **(d)** HR-TEM images of 7% BN/In_2_S_3_. **(e)** Proposed reaction mechanism for photocatalytic selective oxidation of benzyl alcohol over BN/In_2_S_3_ composite. **(f)** Hydrothermal process for the assembly of BN/In_2_S_3_ composite (Meng et al., 2016). The figure is recreated from Meng et al. (2016).

Hydrothermal method is a facile and time-saving way for constructing nanostructures. Starting from hBNNS (hexagonal boron nitride nanosheets), InCl_3_, and TAA (thiacetamide), Meng et al. synthesized the BN/In_2_S_3_ photocatalysts by a one-pot hydrothermal method (Meng et al., 2016) as shown in Figure 1. The hydrothermal assembled BN/In_2_S_3_ composite showed a good interfacial contact between the hBNNS and the spherical-like In_2_S_3_ nanofragments. The incorporation of BN was found to be effective to enhance the separation and transfer efficiencies of the photogenerated electron-hole charge carriers, and therefore improve the photocatalytic activities, where the highest activity was reached at the 7% addition level of BN in the composite. Besides the normal hydrothermal approach, the ionic liquid assisted solvothermal synthesis was also applied for the assembly of the photocatalytic composites BN/Bi_4_O_5_I_2_ (Ji M. et al., 2018) and BN/BiPO_4_ (Chen et al., 2017). In the solvothermal synthesis of BN/Bi_4_O_5_I_2_, the ionic liquid 1-hexyl-3-methylimidazolium iodide ([Hmim]I) served not only as the starting materials, but also as the dispersant to optimize the contact at the interface between the BN and the Bi_4_O_5_I_2_. In another case for BN/BiPO_4_, the ionic liquid 1-decyl-3-methylimidazolium dihydrogen phosphate ([Omim]H_2_PO_4_) was used to assist the solvothermal assembly of the composite with an increased charge transfer efficiency, suppressed hole-electron recombination as well as the enriched active species.

### Ball-milling synthesis

**Figure 2:**
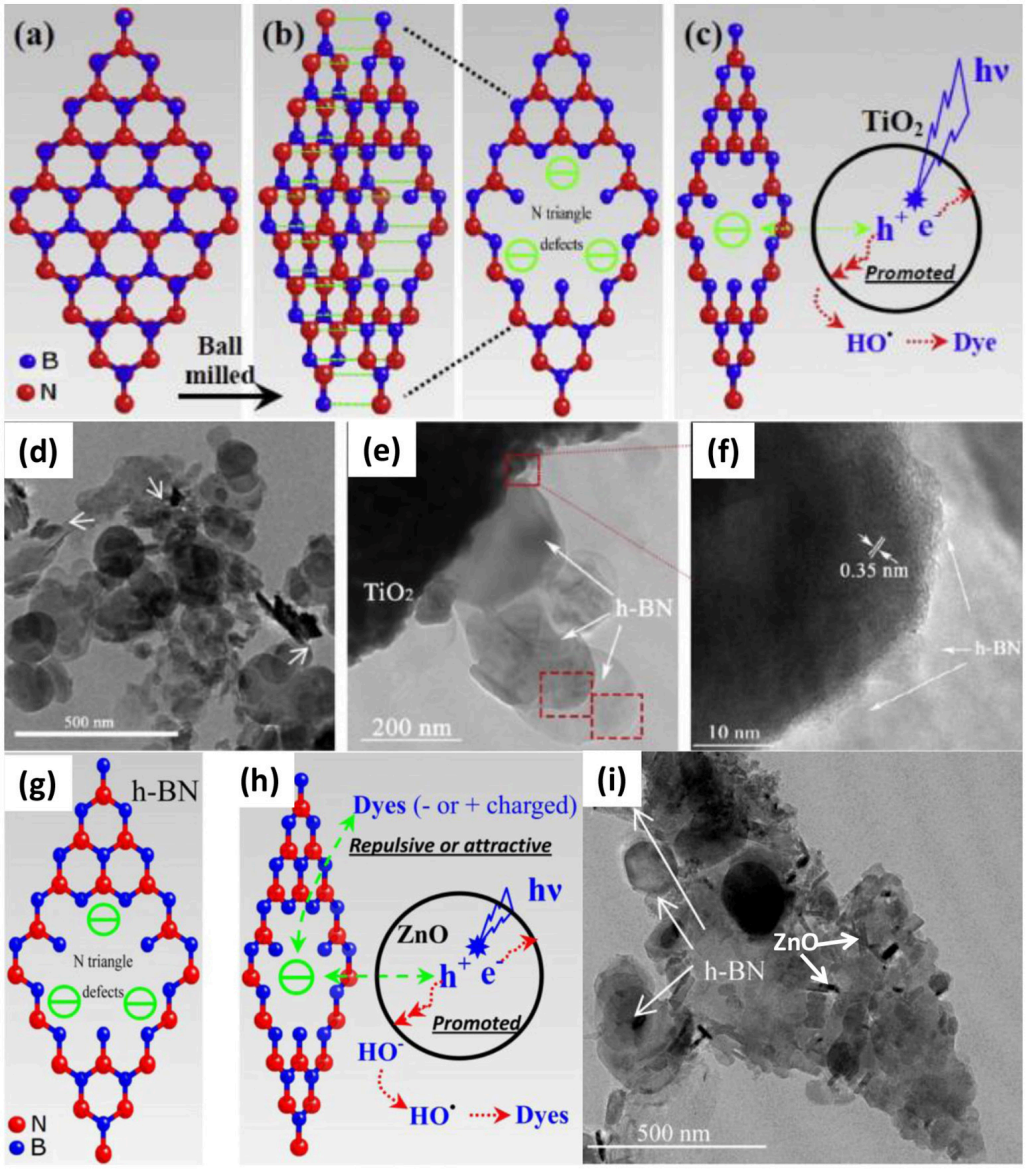
Schematic diagrams of: **(a)** and **(b)** the cleavage of hBN and the structure of N edged triangle defects caused by the ball milling process, and **(c)** the mechanism of the enhanced photocatalytic activity. **(d,e)** TEM and **(f)** HRTEM image of hBN (0.5 wt%)/TiO_2_ prepared by ball milling for 30 min (Fu et al., 2013a). Schematic diagrams of: **(g)** the formation and the structure of N edged triangle defects caused by the ball milling process, and **(h)** the effect of negatively charged hBN on the photocatalytic performance of ZnO. **(i)** TEM images of hBN (1.0 wt.%)/ZnO prepared by ball milling for 20 min (Fu et al., 2013b). The figure is recreated from Fu et al. (2013b).

Ball-milling is a high energy method to introduce numerous defects into the BNNS, which was effective to improve the photocatalytic activities of BN based nanocomposites. Recently, Zhang et al. used the ball-milling method to prepare the Pt/BN/CdS nano-composite photocatalyst (Zhang R. et al., 2015), where the heterogeneous BN/CdS was firstly prepared by a ball milling process, and then the Pt was loaded onto the BN/CdS surface. It was found that the photocatalyst Pt/BN/CdS could exhibit the highly improved photocatalytic activities, and the highest H_2_ evolution rate could reach 17.56 mmol/g/h. The enhanced activity could be ascribed to the heterogeneous design of the photocatalyst, where the BN acted as the hole transfer, while Pt acted as the electron acceptor, both of which may resulted in the effective separation of hole/electron carriers. Most importantly, the ball-milling could enhance the tight contact between the CdS and the BN, facilitating the effective charge transfer. In other BN based nano-composite photocatalysts, Fu et al. synthesized the BN/TiO_2_ nanocomposite via ball-milling method (Fu et al., 2013a), where an optimized ball milling condition (0.5 wt% BN, milling for 30 min) was applied for the synthesis of BN/TiO_2_ nano-composite photocatalyst with 15 and 8 times improvement of efficiency in the degradation of Rhodamine B (RhB) and methylene blue (MB), respectively. Again, the increase of the photocatalytic activities could also be owing to the hole carrier transfer promoting effect of BN (Figures 2a–h). With respect to the BN/ZnO photocatalyst, Fu et al. presented an effective ball milling method (1 wt% BN, milling for 20 min) to assemble the BN/ZnO nanocomposite for the degradation of cationic dyes (Figure 2i), where a degradation efficiency upto ~80% could be achieved for RhB (Fu et al., 2013b). The BN in the BN/ZnO nanocomposite could not only improve the hole carrier transfer, but also provide the active sites for the absorption of the charged dyes via electrostatic forces.

### Calcined synthesis

**Figure 3:**
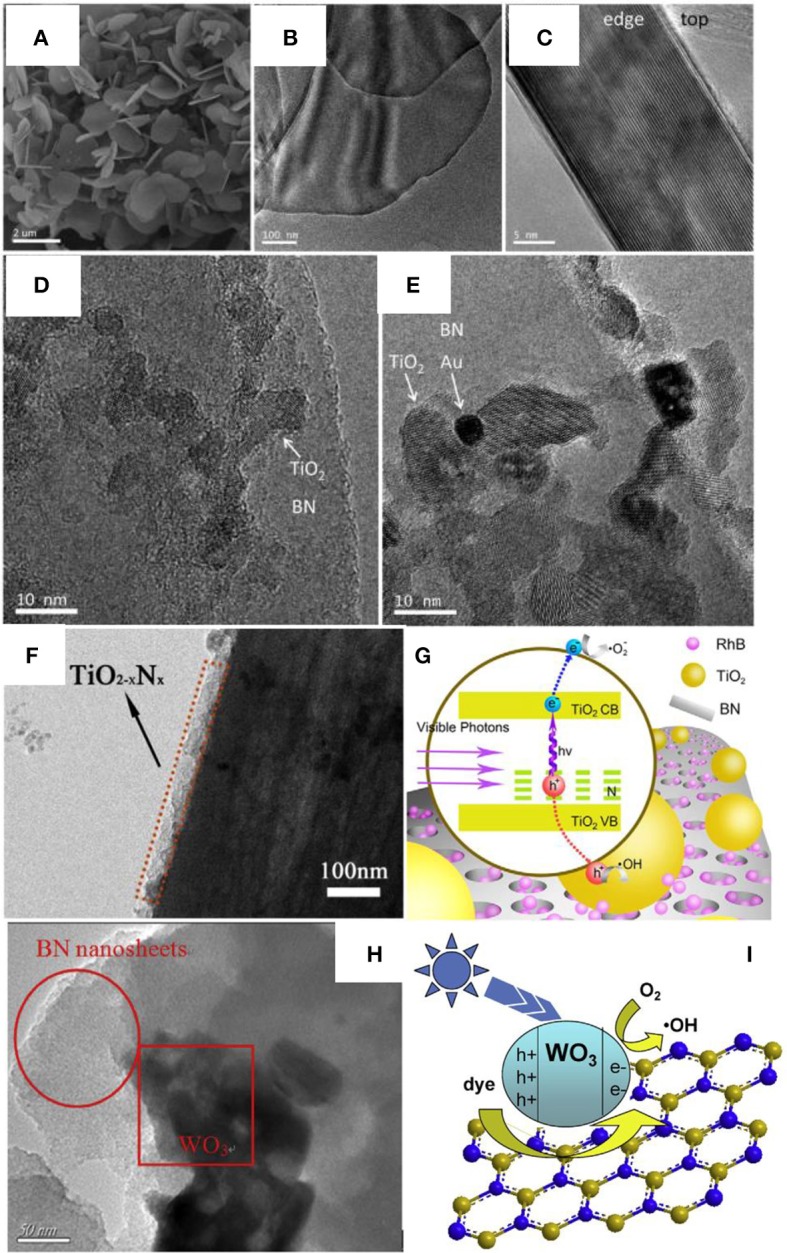
**(A)** SEM, **(B)** TEM, and **(C)** side-view HRTEM images of BN nanosheets, and HRTEM images of **(D)** TiO_2_/BN and **(E)** Au/TiO_2_/BN hybrids (Ide et al., 2014). **(F)** TEM image of the TiO_2−x_N_x_/BN photocatalyst (3:1). **(G)** Mechanism of photocatalysis on RhB under visible light irradiation (Liu et al., 2014). **(H)** TEM image of BN/WO_3_ photocatalyst. **(I)** Proposed mechanisms for the photodegradation of RhB by BN/WO_3_ composites (Xu et al., 2016). The figures are recreated from Ide et al. (2014), Liu et al. (2014), and Xu et al. (2016).

Different from the normal assembly methods, calcination is an effective approach to improve the crystallinity as well as the heterogeneous contact in the nanocomposites, which is critical for the photocatalytic activities. As for the BN based nanocomposite, Ide et al. prepared the Au/TiO_2_/BN hybrid photocatalyst though a calcination method, where the BNNS materials were firstly annealed at 1,200°C for 4 h under N_2_ flow to improve the crystallinity, then the heterostructure Au/TiO_2_/BN was prepared by annealing at 400°C for 2 h under air (Ide et al., 2014). This heterostructure showed high crystallinity and good interface contact (Figures 3A–E), which benefited the nanocomposite with a high visible light-responsive photocatalytic activity (10.3 μmol/h). Within the hybrid structure, the Au could enhance the visible light absorption, the BN could increase the concentration of reactants, and the charge separation could be improved at the BN/TiO_2_ interfaces. Liu et al. prepared the TiO_2_-xN_x_/BN heterostructure via two-step annealing method, where the TiN/BN composite was firstly obtained after annealing at 1,200°C in ammonia, and then the TiO_2_-xN_x_/BN was collected though a calcination at 600°C in air (Liu et al., 2014). As shown in Figure 3F, the nitrogen doped TiO_2_ was uniformly grown on the BN fibers, which should be ascribed to the annealing and calcination process, and the good contact between TiO_2−x_N_x_ and BN on the interfaces, should lead to the high separation efficiency of the photogenerated hole/electron carriers (Figure 3G). Recently, Xu et al. reported the calcination assembly of BN/WO_3_ hybrid photocatalyst (Xu et al., 2016). In the synthesis, the BNNS and WO_3_ nanopowders were firstly mixed and grounded for 30 min, and then the mixture was annealed at 450°C to get the BN/WO_3_ nanocomposite. As shown in Figure 3H, after annealing, the WO_3_ nanoparticles could be tightly adhered onto the BNNS (strong ultrasonic treatment could not peel off the WO_3_ from the BNNS), which should provide the efficient separation of photogenerated electron/hole carriers on the interfaces (Figure 3I), and the 5 wt% BN/WO_3_ showed the best photocatalytic activity 82% for the pollutant RhB, far superior to the single TiO_2_ phase.

### Electrospinning synthesis

**Figure 4:**
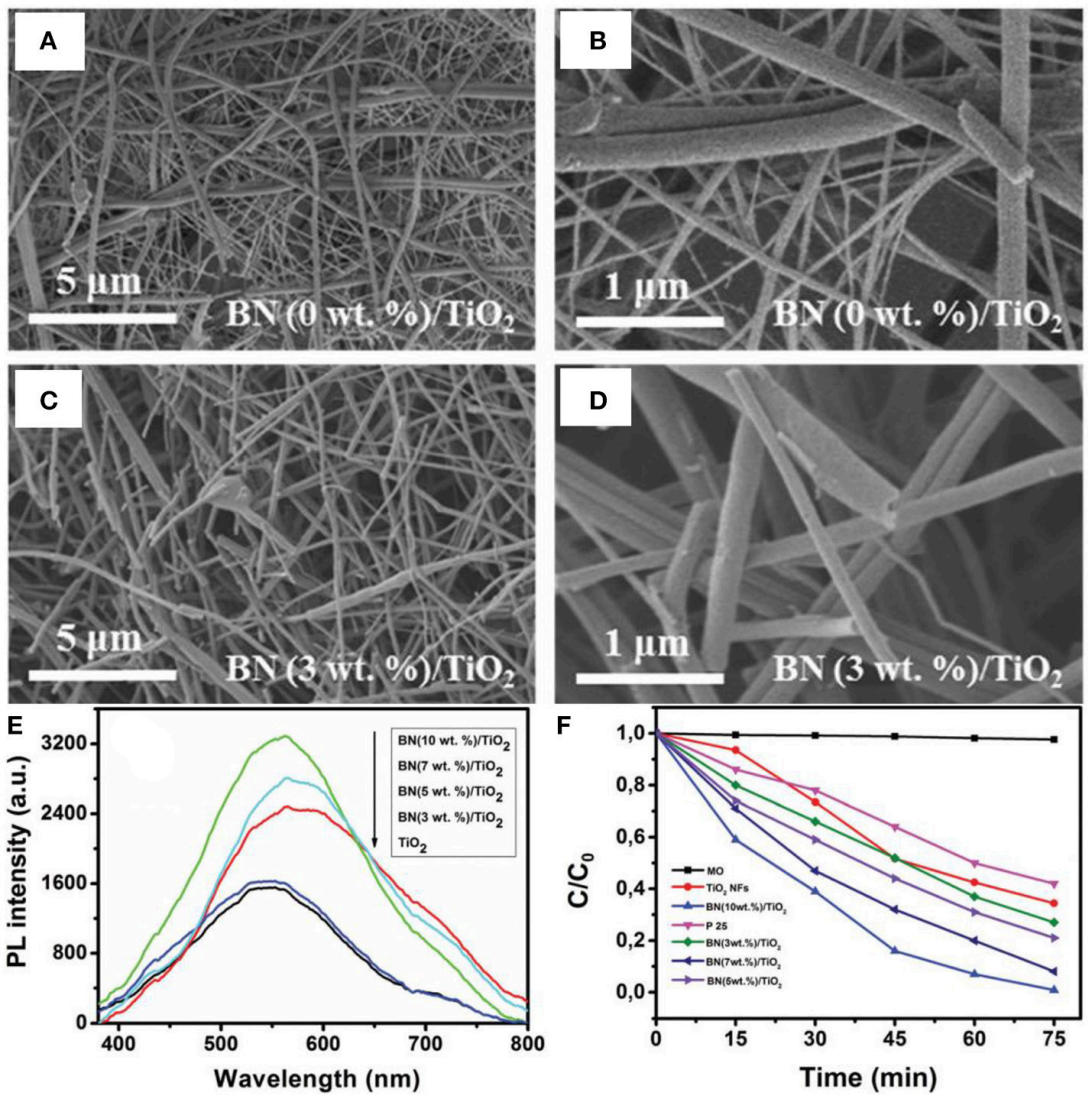
**(A–D)** SEM images of TiO_2_ and BN/TiO_2_ annealed composite nanofibers in air for 4 h at 500°C. **(E)** Photoluminescence of TiO_2_ and BN/TiO_2_ nanofibers annealed in air at 500°C. **(F)** Photodegradation of MO (methyl orange) by P25, TiO_2_ nanofibers, and BN/TiO_2_ composite nanofibers (Nasr et al., 2017). The figure is recreated from Nasr et al. (2017).

In the nanostructure assembly, the electrospinning is an intriguing method that permits the controllable synthesis of nanofibers with designed diameters ranging from nano- to micro-meters, depending on the inherent properties of polymers and/or the processing conditions. For the first time, Nasr et al. prepared the BN/TiO_2_ nanocomposite via electrospinning method for the photodegradation of methyl orange (Nasr et al., 2017). In the electrospinning synthesis, the BNNS and the TiO_2_ precursor were firstly homogenized, and then the mixture was coated onto an aluminum foil using an electrospinning system as the fibers. Finally, the BN/TiO_2_ composite nanofibers were collected and calcined at 500°C in air (Figure 4). As shown in Figures 4A–D, the electrospun nanocomposite photocatalyst showed a morphology with highly interconnected network composed of continuous, randomly oriented nanofibers. The electrospun BN/TiO_2_ nanofibers exhibited the enlarged PL emission (Figure 4E), and was seemed to be related with the oxygen vacancies caused by the BN coupled on the TiO_2_ surface. In addition, the BN/TiO_2_ nanofibers showed higher degradation efficiency (65%) for the methyl orange than that (60%) of the commercialized P25 photocatalyst (Figure 4F). Except for the BN based nanocomposite, the electrospinning method could also been applied for other photocatalysts. For example, the ZnO/TiO_2_/ZnO or TiO_2_/ZnO/TiO_2_ heterostructured photocatalysts could be fabricated with an electrospinning and a followed atomic layer deposition (ALD) method (Kayaci et al., 2014); the TiO_2_-polyamide-12 nanocomposites could be electrospun synthesized for clear water (Cossich et al., 2015); the graphene oxide segregated TiO_2_ nanofibers could be got by electrospinning for visible light photocatalysis (Zhang et al., 2017); the MgO nanofibers could be synthesized by electrospinning for photo induced dye degradation (Mantilaka et al., 2018). Besides, the BiOCl/TiO_2_ (Wang K. et al., 2015), Ag/LaFeO_3_ (Li et al., 2016), Ag/Ga_2_O_3_ (Han et al., 2017), BiOCl_x_/BiOBr_y_/BiOI_z_ (Zhang et al., 2016), graphene-ZnO (An et al., 2014), Ag@AgCl (Zhou et al., 2016), BiOBr (Babu et al., 2016), and mesoporous TiO_2_ (Fu et al., 2014) photocatalysts could also been assembled by the electrospinning methods. In all, the electrospinning method is a facile and controllable approach for the assembly of micro/nanofiber structured photocatalysts, and the core-shell or hierarchical structures could also be obtained via the combination of electrospinning and ALD/hydrothermal methods.

### Other methods

**Figure 5:**
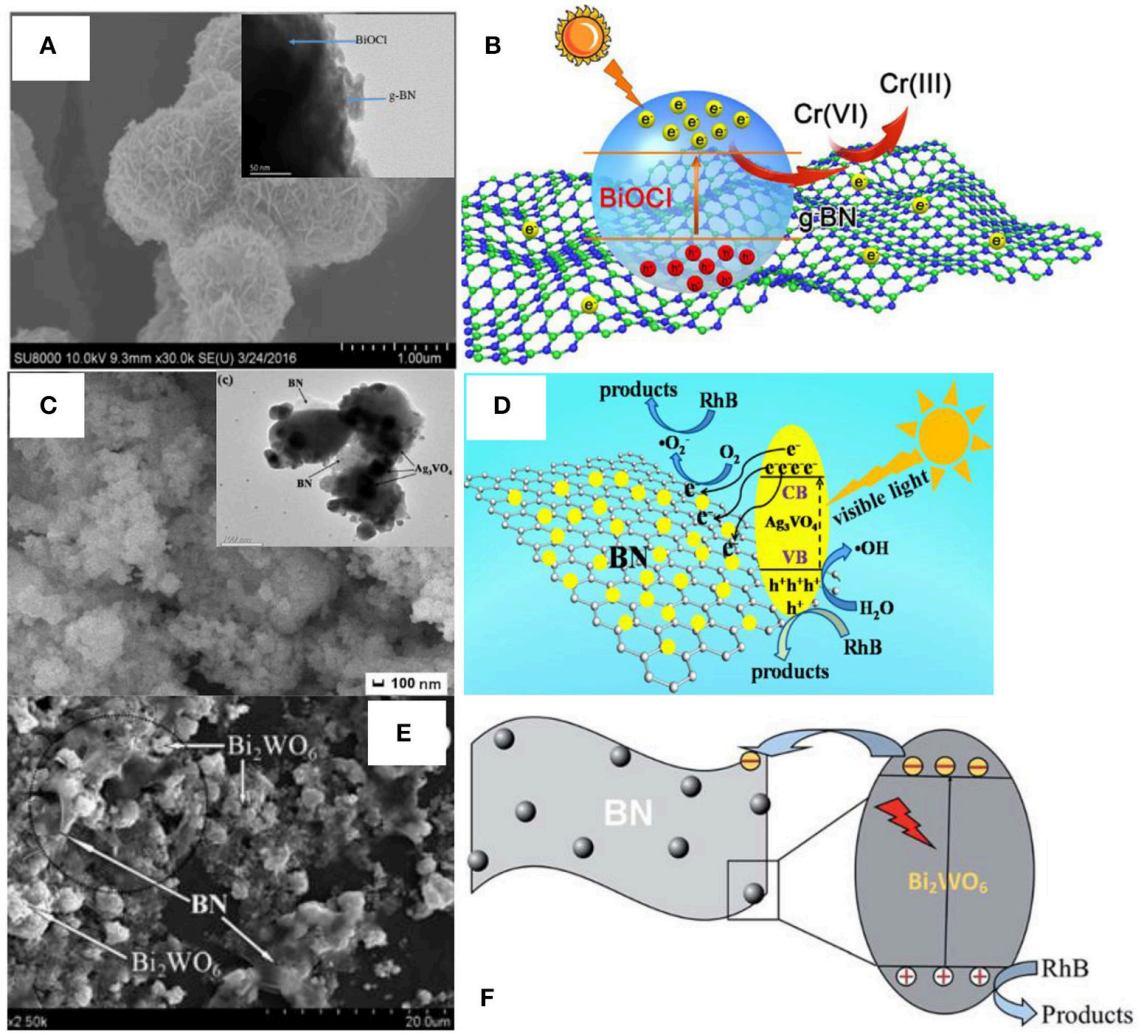
**(A)** FESEM image of the 1% BN/BiOCl composite. Inset is the corresponding TEM image. **(B)** Proposed mechanism for the photoreduction of Cr (VI) over the 1% BN/BiOCl composite (Xu et al., 2017a). **(C)** FESEM image of the 3 wt% hBN/Ag_3_VO_4_. Inset is the corresponding TEM image. **(D)** Proposed mechanism for the RhB photodegradation over the catalyst hBN/Ag_3_VO_4_ (Lv et al., 2016). **(E)** SEM image for the BN/Bi_2_WO_6_ photocatalyst. **(F)** Proposed mechanism for the RhB photodegradation over the photocatalyst BN/Bi_2_WO_6_ (Li et al., 2015). The figures are recreated from Xu et al. (2017a), Lv et al. (2016), and Li et al. (2015).

Apart from the above methods, the scientists also proposed the other approaches for the assembly of BN based photocatalysts, including the *in-situ* precipitation (Lv et al., 2016), impregnation (Li et al., 2015), the microwave-assisted (Xu et al., 2017a), and the water bath synthesis methods (Chen et al., 2014). As shown in Figure 5, the microwave assisted method was used to synthesize the BN/BiOCl composite (Figures 5A,B) where the BN modified BiOCl microspheres were formed with an enhanced photoreactivity for the reduction of Cr(IV) with visible-light irradiation. The *in-situ* precipitation was used to prepare the hBN/Ag_3_VO_4_ photocatalyst where the Ag_3_VO_4_ photocatalyst was stabilized via wrapping with the 3D hBN hierarchical structure during the *in-situ* precipitation process, which promoted the degradation ability of RhB under the visible light irradiation (Figures 5C,D). The impregnation method has been used to fabricate the BN/Bi_2_WO_6_ photocatalyst (Figures 5E,F), where the BN few-layer structures were loaded onto the Bi_2_WO_6_ catalyst by refluxing the mixture in the ethyl alcohol for 11 h, and the 3 wt% BN/Bi_2_WO_6_ product exhibited the best visible-light photocatalytic activities for the degradation of RhB. The water bath method could be used to prepare the BN/AgBr photocatalyst (Chen et al., 2014), where a water bath process was used to synthesis the AgBr catalyst on the BN substrates, which gave rise to a high catalytic activity for the degradation of MO under visible light irradiation. In a short summary, the low-energy methods, including the electrospinning, *in-situ* precipitation, and impregnation are more flexible for the preparation of composite catalysts with various nanostructures, but the weak interface interactions will restrict the performance enhancement, and a followed annealing process may be necessary for the high catalytic activities. In contrast, the high-energy methods, including the hydrothermal, ball milling, calcination, microwave-assisted, and the water bath could result in the strong interface interactions between the BN and the catalysts, thereby leading to the excellent photocatalytic activities, although the flexibility for the nanostructures are restricted.

## Effect of BN in the photocatalysts

Due to the wide band gap (~5.5 eV), the hBN material could only take effect in the ultraviolet wavelength for the photocatalysis (Ji H. et al., 2018; Xing et al., 2018; Zhang X. et al., 2018). Despite the wide band gap, the hBN nanostructures were intensively applied for the photocatalytic nanocomposites, owing to its high thermal/chemical stability and the hole promoting ability. In the nanocomposite for photocatalysis, the hBN could play different roles for improving the photocatalytic activities. As summarized in Table 1, within the heterogeneous compound, hBN could be used as carrier promoter, *h*^+^ transfer channel, and could be used to enhance the visible light/electron absorption, form the B-O-Ti dangling bond, and generate the •OH and *h*^+^.

**Table 1:** Effect of hBN in the photocatalytic composites.

**Catalysts**	**hBN morphology**	***E*_g_/eV^a^**	**Effect of hBN**	**References**
hBN/Bi_2_WO_6_	Nanosheets	2.68/2.90	*h*^+^ Promoter, *A*_vis_↑	Li et al., 2018
hBN/gC_3_N_4_	Nanosheets	2.42/2.56	*h*^+^ Transfer channel, *A*_vis_↑	Jiang et al., 2018
hBN/Bi_4_O_5_I_2_	Nanosheets	2.23/2.23	Electron absorption	Ji M. et al., 2018
hBN/TiO_2_	Porous nanosheets	2.95/3.13	B-O-Ti bonding, *A*_vis_↑	Liu et al., 2017
BN-DS-7	Nanoplate	3.94	Generate •OH and *h*^+^	Guo et al., 2018

a *The band gap (Eg) for the composite (left) and the unmodified catalyst (right). The h^+^ is the hole carrier. A_vis_ is the visible light absorption. The symbol ↑ represents an enhancement*.

### Photogenerated carrier transfer promoter

**Figure 6:**
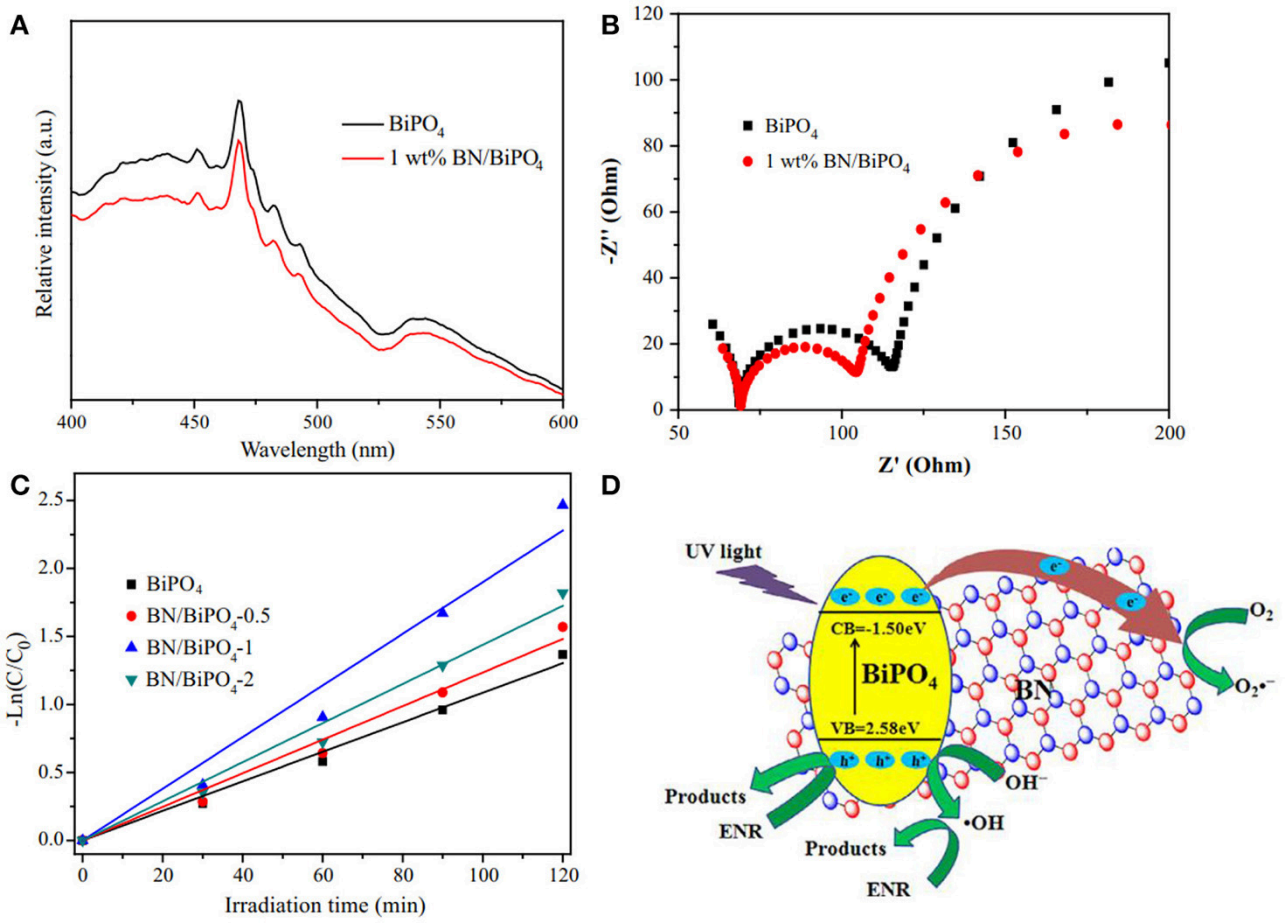
**(A)** Photoluminescence spectra of the obtained pure BiPO_4_ and 1 wt% BN/BiPO_4_ composites. **(B)** Electrochemical impedance spectroscopy (EIS) Nyquist plots of the sample electrodes of pure BiPO_4_ and 1 wt% BN/BiPO_4_ composites. **(C)** Kinetic fit for the degradation of ENR with the pure BiPO_4_ and BN/BiPO_4_ samples. **(D)** Proposed mechanism for the separation and transfer of photogenerated charges of BN modified BiPO_4_ materials under UV light irradiation (Chen et al., 2017). The figure is recreated from Chen et al. (2017).

Reducing the recombination rate of the photogenerated h^+^/e^−^ carriers is critical for the improvement of photocatalytic activities. Chen et al. reported the modification of bismuth phosphate via the hBN nanosheets (Chen et al., 2017). After the modification, the PL intensity became weaker than the pure bismuth phosphate (Figure 6A), which was ascribed to the reduction of the recombination of the photogenerated h^+^/e^−^ carrier pairs, and the smaller charge transfer resistance (Figure 6B) revealed the enhanced photocatalytic efficiency of the hBN modified composites which was then proved by the kinetic analysis of the photodegradation (Figure 6C). As suggested in Figure 6D, the active species •OH and ${O_{2}^{•}}^{-}$ could be generated on the bismuth phosphate by the redox reaction OH^−^ + h^+^ → •OH (valence band, VB) and O_2_ + e^−^→${O_{2}^{•}}^{-}$ (conduction band, CB). Due to the efficient carrier (h^+^/e^−^) transfer from bismuth phosphate to the hBN, the recombination of h^+^-e^−^ pairs was reduced, and more active •OH and ${O_{2}^{•}}^{-}$ could be formed to enhance the photocatalytic efficiencies. Such effect in enhancing the carrier transfer rate and reducing the recombination rate of carrier pairs could also be found in the photocatalyst hBN/Ag_2_CO_3_ (Wang J. et al., 2015).

Moreover, in the composite hBN/TiO_2_, it was found that the hBN could behave as an efficient hole carrier transfer promoter via the negatively charged hBNNS (hBN nanosheet) surface after a ball-milling process or though the intrinsic electrostatic potential of hBNNT (hBN nanotube; Tang et al., 2010; Fu et al., 2013a). In the hBN/ZnO photocatalyst, the hBNNS could also serve as the hole transfer promoter via the negatively charged surface cunducted by the ball milling (Fu et al., 2013b).

### Enhancing the visible light absorption

Due to the wide band gap (E_g_ > 2.0 eV), the low absorption of visible light in the photocatalysts (e.g., TiO2, *E*_*g*_ = 3.2 eV) restricts the photodegradation applications under the sun light. The hBN incorporation was found to be effective in reducing the band gap to improve the visible light absorption of photocatalysts, which resulted in the enhancement of visible light-responsive photocatalytic activities (Table 1). As depicted in Table 1, the addition of hBN in the photocatalysts could reduce the Eg of Bi_2_WO_6_, gC_3_N_4_, and TiO_2_ from 2.90, 2.56, and 3.13 eV to 2.68, 2.42, and 2.95 eV, respectively, which resulted in the increase of visible light absorption. However, in the hBN/BiPO4 composite, the electronic structure of BiPO4 could not be influenced by the hBN (~1 wt%; Chen et al., 2017), and in another case for hBN/Bi_2_WO_6_, the band gap was widened by the hBN incorporation (Li et al., 2015). The different behaviors of hBN in tailoring the electronic structure of the photocatalysts should be ascribed to the different strain accumulation on the interface between the hBN and the photocatalyst, since the strain could play a vital role in tuning the band gap of semiconducting materials (Ishikawa et al., 2003; Minot et al., 2003; Biele et al., 2018; Song et al., 2018; Wang et al., 2018; Zhang Y. et al., 2018). It should be noticed that the composite prepared with low-temperature methods, e.g., impregnation, always possessed unchanged or widened *E*_g_, whilst the high-temperature methods, e.g., hydrothermal, annealing always resulted in narrowed *E*_g_. Therefore, it should be careful to tune the interface adhesion via controlling the fabrication methods for successfully reducing the band gap of the hBN incorporated composite, and achieving the high visible light absorption.

### Dangling bond

**Figure 7:**
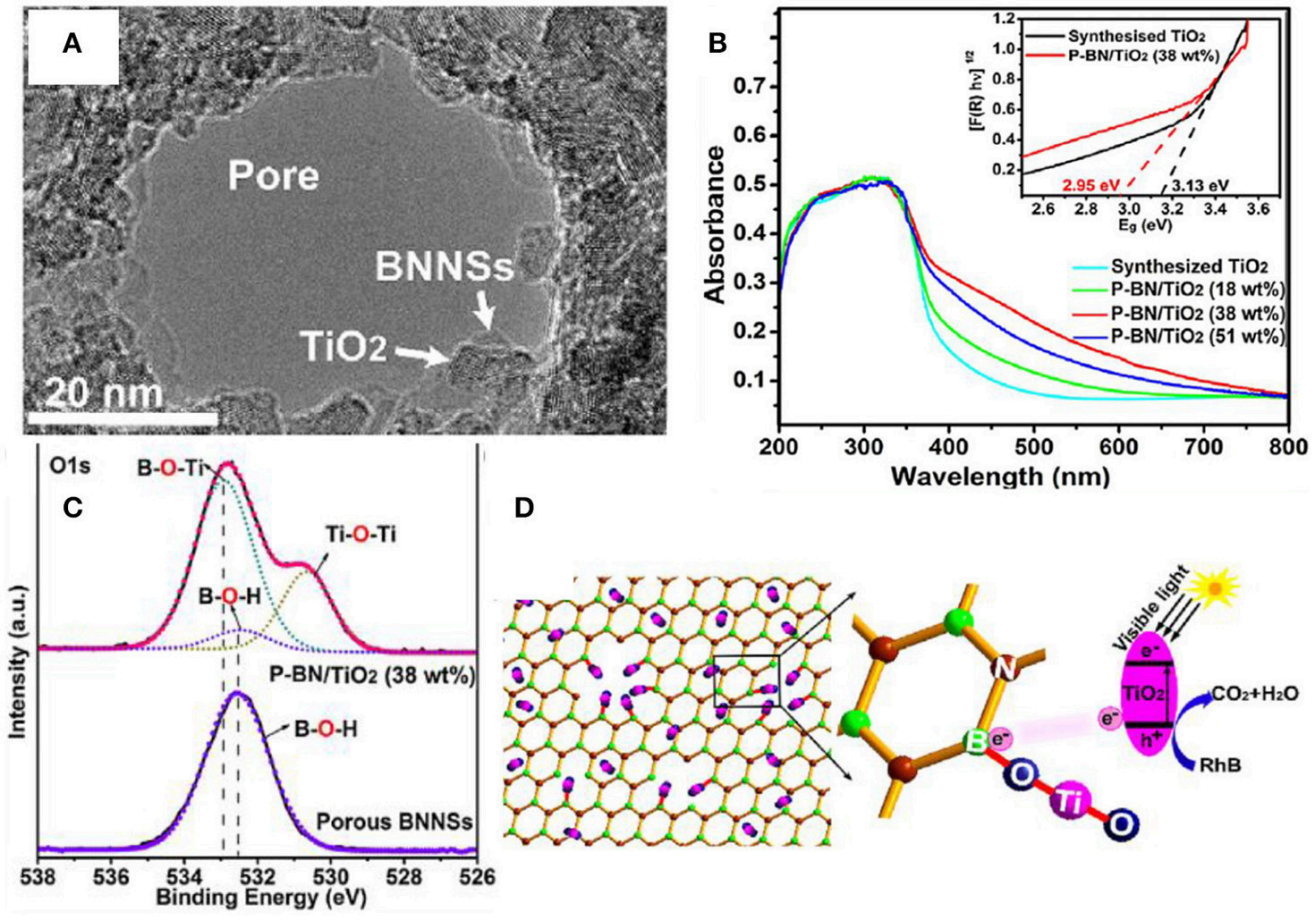
**(A)** TEM image for an hBN hole decorated by TiO nanoparticles. **(B)** UV–vis absorption spectra of synthesized TiO_2_ and different porous BN/TiO_2_ hybrid nanosheets (for comparison). The inset shows the relationship between the transformed Kubelka-Munk function vs. the light energy for synthesized TiO_2_ and porous BN/TiO_2_ (38 wt%) hybrid nanosheets. **(C)** High resolution XPS scan of the O 1s for the hBN/TiO_2_ composite. **(D)** Schematic diagram for the photocatalytic reaction of RhB by porous BN/TiO_2_ hybrid nanosheets with new chemical bonding specie B-O-Ti under simulated visible light (Liu et al., 2017). The figure is recreated from Liu et al. (2017).

Introducing the dangling bond in the photocatalysts is an effective approach for improving the visible light absorption and enhancing the photocatalytic activities under the visible light irradiation (Hirsch and Hauke, 2017; Chen H. et al., 2018; Chen T. et al., 2018; Ma et al., 2018). With respect to the hBN incorporated composites, it is possible to form the dangling bond at the edge of the pores of hBNNS. As demonstrated in the porous hBN/TiO_2_ composites (Liu et al., 2017), the dangling bond B-O-Ti at the open edges of the hBN pores could cause the energy rearrangement of the composite, and thus reduce the band gap from 3.13 to 2.95 eV, and improve the absorption of visible light (Figure 7).

In short, the effect of hBN in the catalytic composites discussed in literatures is mainly focused on the decrease of *E*_g_, the hole carrier promoting, and the B-O-Ti dangling bond. However, the *E*_g_ tuning mechanisms and the molecular dynamics for the hole promoting are rarely reported.

## Conclusions

In summary, the visible light-responsive hBN incorporated photocatalytic composites were fully reviewed based on the recent published works. Firstly, the preparation methods, including the hydrothermal, ball milling, calcined, electrospinning, *in-situ* precipitation, impregnation, microwave-assisted, and water bath methods, were discussed with respect to the photocatalytic activity, interface adhesion, crystallinity as well as the cost. Among these approaches, the hydrothermal and calcined methods showed the high crystallinity as well as the good interface adhesion, which gave rise to the high photocatalytic activities, while the electrospinning method exhibited the advantages in flexibility and large aspect ratio of the catalysts. The other methods also represented the facile approaches for the fabrication of hBN incorporated photocatalysts. Regarding the activity enhancing mechanism through the incorporation of hBN nanostructures, the possible hypothesis could be the photogenerated carrier (hole/free electron) transfer promoter (e.g., hBN/BiPO_4_ and hBN/Ag_2_CO_3_), the visible light absorption enhancement (e.g., hBN/BiPO_4_ and hBN/Bi_2_WO_6_), and the dangling bond formed at the interface (e.g., hBN/TiO_2_). Therefore, the optimized hBN incorporated photocatalysts should have the strong adhesion at the interface as well as the high crystallinity, which could result in the reduced recombination rate of the photogenerated carrier pairs, the improved visible light absorption, and thus lead to the enhanced photocatalytic activities under the visible light irradiation.

## Outlook

In order to promote the photocatalytic activities of the hBN incorporated composites, the following investigations could be taken into consideration in the future:

- Band gap engineering for the hBN used in the composites (Ba et al., 2017), e.g., doping, strain engineering.
- 3D aligned hBN nanostructure (nanotube, nanosheets) for the assembly of the composites with excellent interface adhesion (Wu et al., 2016), e.g., ice template (Gao et al., 2014), biomimetic assembly.
- Doped hBN for the band gap reduction of the composites (Weng et al., 2017), e.g., cations, anions doping.
- Gradient band gap design at the interface of the composites (Lan et al., 2016; Mitsutaro et al., 2016), e.g., hBN/VO_2_/TiO_2_ heterostructures.
- SPR enhanced activities for the hBN incorporated composites via modification with metal nanoparticles (Wu et al., 2015, 2016), e.g., Ag, Au modification on the surface.

## Author contributions

NW, GY, HW, RS, and C-PW proposed and wrote the manuscript.

### Conflict of interest statement

The authors declare that the research was conducted in the absence of any commercial or financial relationships that could be construed as a potential conflict of interest.
